# The complete chloroplast genome sequence of *Ranunculus pekinensis* (L. Liou) Luferov (Ranunculaceae), a species endemic to China

**DOI:** 10.1080/23802359.2022.2073841

**Published:** 2022-05-11

**Authors:** Xuehua Liu, Wenli Yang, Danke Zhang, Hao Liu, Yueming Cui, Lei Wang, Hongyu Hu, Yue Yin, Gangmin Zhang

**Affiliations:** a Laboratory of Systematic Evolution and Biogeography of Woody Plants, School of Ecology and Nature Conservation, Beijing Forestry University, Beijing, People’s Republic of China; b College of Landscape Architecture and Tourism, Hebei Agricultural University, Baoding, People’s Republic of China; c College of Horticulture and Plant Protection, Henan University of Science and Technology, Luoyang, People’s Republic of China

**Keywords:** *Ranunculus pekinensis*, complete chloroplast genome, phylogenetic analysis, Ranunculaceae

## Abstract

*Ranunculus pekinensis* (L. Liou) Luferov 1997, a perennial aquatic herb, is endemic to Beijing, China and has high water quality requirements. Because its habitat is under great threat and its population is declining, it is now listed as a national protected plant in China. To provide genomic resources for future research of this endangered species, the complete chloroplast genome sequence of *R. pekinensis* was assembled and annotated for the first time. The complete chloroplast genome sequence was 156,139 bp in length, containing a large single copy region (LSC) of 85,430 bp and a small single copy region (SSC) of 19,970 bp, which were separated by a pair of 25,367 bp inverted repeat regions (IRs). The complete chloroplast sequence contained 112 unique genes, including 30 tRNA, 4 rRNA, and 78 protein-coding genes. The overall guanine-cytosine (GC) content of the chloroplast genome was 37.8%, and the GC contents of the LSC, SSC, and IR regions were 36.0%, 31.3%, and 43.5%, respectively. Phylogenetic analysis with the reported chloroplast sequences showed that *R. pekinensis* was closely related to *R. bungei* Steud. 1841, both of which belonged to *Ranunculus* Sect. *Batrachium* DC. 1817. These data will provide essential resources regarding the evolution and conservation of *R. pekinensis*.

*Ranunculus* L. 1753 (Ranunculaceae) is widely distributed on all continents except for Antarctica, mainly in north temperate regions, with about 550 species in total (Wang and Michael 2001). *Ranunculus pekinensis* (L. Liou) Luferov 1997, a perennial aquatic herb, is endemic to Beijing, China, and grows in valley streams at altitudes of 80–1200 m (Wu et al. 2014). This species belongs to *Ranunculus* Sect. *Batrachium* DC. 1817 and has high water quality requirements (Han et al. 2020). Over the past 30 years, the continuous drought in this area has left many streams depleted of water, which has led directly to habitat loss. Water pollution caused by domestic waste and herbicides also seriously threatens the survival and reproduction of *R. pekinensis* (Wu et al. 2014). Recently, *R. pekinensis* was listed as a national protected plant species. Therefore, it is vital to implement effective science-based protection measures to expand the population size.

Chloroplast in plant cells is a double membrane-bounded organelle that plays vital metabolic roles including photosynthesis, amino acid and lipid synthesis. In most plant species, the chloroplasts (cp) have a circular genome, varying in size from 72 to 217 kb and containing about 130 genes. And the chloroplast genome has a quadripartite structure in which a pair of inverted repeats (IRs) region separates a large single copy (LSC) from small single copy (SSC) regions in most species. Numerous mutational events take place in chloroplast genomes, including substitutions, insertions and deletions (InDels), inversions, genome rearrangements, and translocations. Polymorphism in chloroplast genomes has been exploited to resolve taxonomic and phylogenetic discrepancies. Moreover, it is also useful for development of DNA barcodes, population genetics and evolutionary studies (Mehmood et al. 2020). However, to date, no chloroplast genome resources have been available for *R. pekinensis*. In this study, the complete chloroplast genome of *R. pekinensis* was assembled to provide a genetic foundation for further research.

Fresh young leaves of *R. pekinensis* were collected from Yudu Mountain in Beijing, China (N 40°33′7.38″, E 115°52′43.73″), the specimens were deposited at the Herbarium of Beijing Forestry University (http://bjfc.bjfu.edu.cn, Liangcheng Zhao: lczhao@bjfu.edu.cn) under voucher number LXH001. The CTAB method (Doyle and Doyle 1987) was used to extract the total genome DNA. Then, an Illumina HiSeq 4000 platform at Novogene (http://www.novogene.com, China) was used to perform 2 × 150 bp pair-end sequencing. The Map to Reference function in Geneious Prime (Kearse et al. 2012) was used to exclude nuclear and mitochondrial reads using the published plastid genome of *R. bungei* Steud. 1841 as a reference (Accession no. MK253468). The filtered chloroplast reads were then de novo assembled (with ‘Low Sensitivity/Fastest’ sensitivity) and concatenated into larger contigs using the Repeat Finder function in Geneious Prime. The raw reads were again mapped to the larger contigs (with the Map to Reference function) to extend their boundaries until all contigs could be concatenated into one contig. Gaps were bridged using the Fine Tuning function of Geneious Prime. The IR region was determined using the Repeat Finder function in Geneious Prime. The mean coverage depth of the assembly was 765×. The assembled chloroplast sequence was annotated using the Plastid Genome Annotator (PGA, Qu et al. 2019) and verified by Geneious Prime. The final *R. pekinensis* chloroplast genome sequence was then submitted to GenBank under the accession number of OK166810.

The chloroplast genome of *R. pekinensis* was 156,139 bp in length, containing a large single copy region (LSC) of 85,430 bp and a small single copy region (SSC) of 19,970 bp, which were separated by a pair of 25,367 bp inverted repeat regions (IRs). The chloroplast genome sequence contained 112 unique genes including 30 tRNA genes, 4 rRNA genes, and 78 protein-coding genes. The overall guanine-cytosine (GC) content of the chloroplast genome was 37.8%, and the GC contents of the LSC, SSC, and IR regions were 36.0%, 31.3%, and 43.5%, respectively.

To understand the phylogenetic relationship between *R. pekinensis* and its related taxa, the complete chloroplast genome sequences of nine *Ranunculus* species and seven other species in Ranunculaceae were downloaded from the NCBI GenBank. All sequences were aligned using MAFFT (Katoh et al. 2017). The maximum likelihood (ML) method was used for phylogeny reconstruction. The sequence alignment was performed as described by Liu et al. (2018). Then, the ML tree was constructed using IQ-TREE software (Kalyaanamoorthy et al. 2017; Hoang et al. 2018). The phylogenetic analysis showed that all *Ranunculus* species clustered together and were closely related to *Oxygraphis* Bunge 1835. They all belonged to the Trib. Ranunculeae of Ranunculaceae. In addition, *R. pekinensis* and *R. bungei* were united with strong support (ML BP = 100), and were robustly nested in the clade of *Ranunculus* (Figure 1). This result was consistent with previous studies (Khatere et al. 2010; Gerhard and Alexander 2017), supporting the conclusion that *Batrachium* (DC.) Gray 1821 should be treated as a section of the genus *Ranunculus*. These data will provide essential resources regarding the evolution and conservation of *R. pekinensis*.

**Figure 1.**
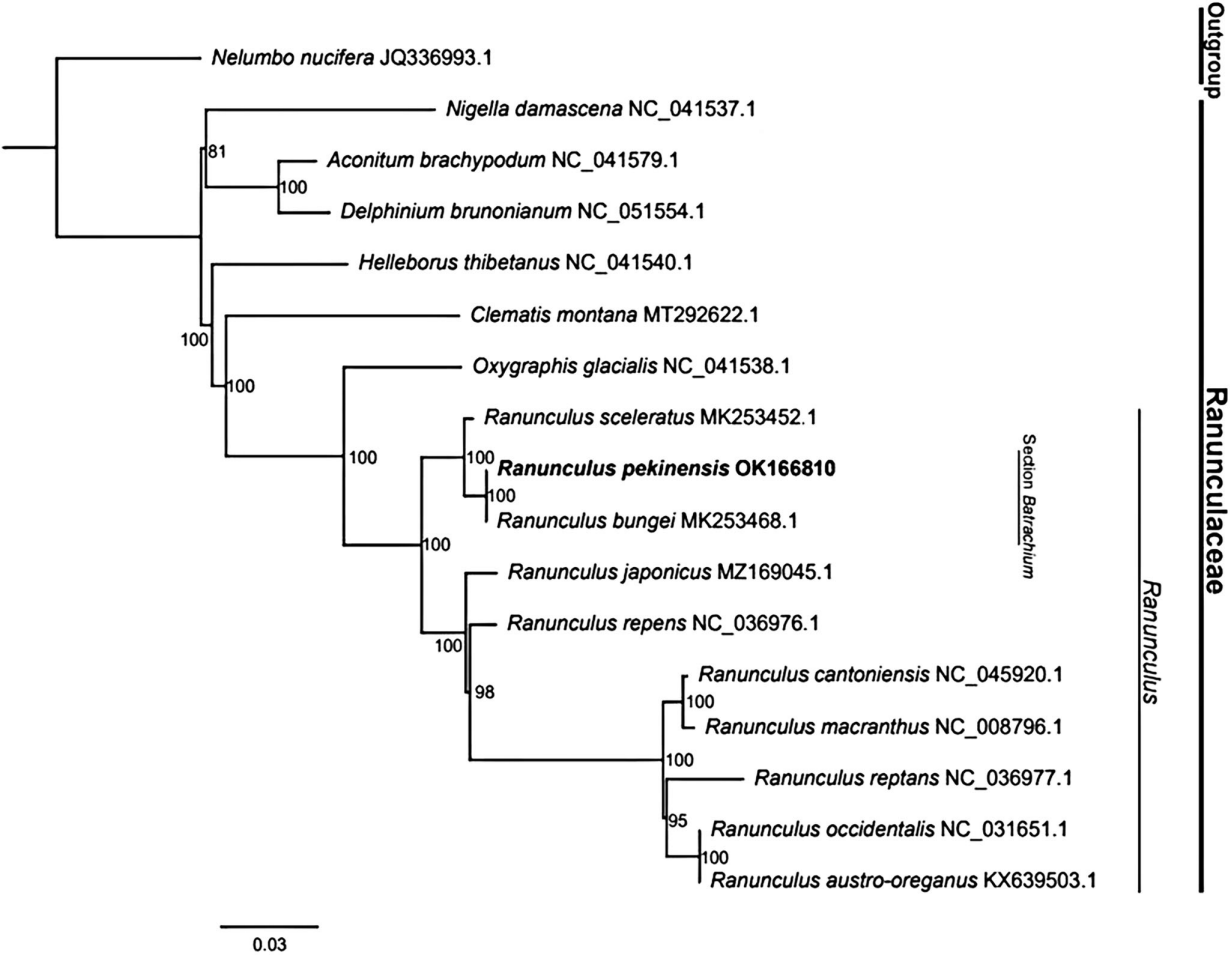
Phylogram of maximum likelihood analysis of 17 species based on chloroplast genome sequences.

## Data Availability

The genome sequence data that support the findings of this study are openly available in GenBank of NCBI at (https://www.ncbi.nlm.nih.gov/) under the accession no. OK166810. The associated BioProject, SRA, and BioSample numbers are PRJNA770464, SRS10552956, and SAMN22218715 respectively.
